# The development and evaluation of a tublysine-based antibody-drug conjugate with enhanced tumor therapeutic efficacy

**DOI:** 10.3389/fphar.2025.1532104

**Published:** 2025-02-10

**Authors:** Huihui Guo, Hongsheng Xie, Yuanyuan Huang, Junxiang Jia, Xiangfei Kong, Qingliang Yang, Shun Gai, Wenjun Li, Lu Bai, Lingli Zhang, Xiaoxiao Chen, Zhicang Ye, Hangbo Ye, Linyao Zhao, Yifang Xu, Yong Du, Xiuzhen Zhang, Miaomiao Chen, Xiaomai Zhou, Robert Y. Zhao

**Affiliations:** Hangzhou DAC Biotechnology Co., Ltd., Hangzhou, Zhejiang, China

**Keywords:** Tub114, DX126-262, tubulysin B analog, target-specific delivery, HER2, therapeutic window, ADC

## Abstract

Antibody-drug conjugates (ADCs) offer targeted cancer therapy by delivering cytotoxic agents directly to tumor cells. However, challenges such as relapse, resistance, and diverse patient needs drive ongoing innovation in ADC development. Exploration of new small-molecule toxins with unique antitumor and toxicity profiles is crucial. Tub114, a novel Tubulysin B analog with a hydrophilic ethylene glycol moiety, has been conjugated to the anti-HER2 antibody DX-CHO9, forming the ADC DX126-262. This study examines the efficacy, pharmacokinetics, and safety profile of DX126-262, with a focus on Tubulysin-associated liver toxicity. *In vivo* efficacy was assessed using three HER2-positive tumor models, with dose-dependent tumor growth inhibition compared to established treatments. Pharmacokinetic studies were conducted in cynomolgus monkeys across a dosing range (3–30 mg/kg) to compare clearance and stability with Kadcyla and Enhertu. Acute toxicity assays were conducted in mice (75 and 150 mg/kg doses), and repeated-dose toxicity was evaluated over five doses, administered every 3 weeks in rats and cynomolgus monkeys. DX126-262 demonstrated significant and dose-dependent tumor growth inhibition across HER2-positive models, with superior antitumor efficacy compared to Kadcyla and comparable efficacy to Enhertu *in vivo* studies. In pharmacokinetic studies, DX126-262 exhibited a clearance rate similar to Enhertu and enhanced stability compared to Kadcyla. Acute toxicity assays revealed reduced hepatotoxicity at doses of 75 and 150 mg/kg in mice, with improved tolerance. In repeated-dose toxicity studies, DX126-262 was well tolerated in rats at doses up to 200 mg/kg, with the highest non-severely toxic dose (HNSTD) established at 100 mg/kg. In cynomolgus monkeys, DX126-262 demonstrated superior hepatotoxic tolerability without significant bone marrow suppression, with an HNSTD of 30 mg/kg. DX126-262, incorporating Tub114, a novel Tubulysin B analog, effectively mitigates the inherent hepatotoxicity associated with Tubulysin compounds while maintaining strong antitumor efficacy. These findings suggest that DX126-262 could serve as a safer and more effective option for HER2-targeted cancer therapy, warranting further clinical studies to confirm its therapeutic potential.

## Introduction

Antibody-drug conjugates (ADCs) combine the tumor-targeting specificity of antibodies with the potent cytotoxicity of payloads, providing a broader therapeutic window compared to traditional chemotherapy. Small molecule toxins, used as payloads, exert their effects through different mechanisms, including the disruption of DNA replication and repair, modulation of microtubule dynamics, and other cellular processes, ultimately inhibiting mitosis and inducing cell death (Conilh et al., 2023; Wang et al., 2023). Due to differences in their mechanisms and physicochemical properties, these toxins exhibit distinct biological profiles.

HER2 (human epidermal growth factor receptor 2) is one of the most frequently chosen targets in ADC research. Small molecules such as DM1, MMAE, MMAF, Dxd, and PBD have been explored at different stages of preclinical and clinical development to improve therapeutic efficacy or expand patient indications (Li et al., 2020; Tymon-Rosario et al., 2021; Yin et al., 2023). Currently, the ADC ado-trastuzumab emtansine (Kadcyla), which uses DM1 as a microtubule inhibitor payload, and trastuzumab deruxtecan (Enhertu), with Dxd as a topoisomerase inhibitor payload, are widely used in HER2-targeted therapies.

Kadcyla is used in the treatment of HER2-positive breast cancer. As a first-generation ADC, it has a limited efficacy window. The maximum tolerated dose (MTD) is 3.6 mg/kg every 3 weeks (Q3W), the same as the dose used for advanced breast cancer (Krop et al., 2010; Lambert and Chari, 2014; de Goeij and Lambert, 2016). The most serious adverse events include hepatotoxicity and hematologic toxicity, both of which, along with cardiac toxicity, are included in its black box warning (Nguyen et al., 2023). Hepatotoxicity typically presents as an asymptomatic, transient increase in serum transaminase levels (Ma et al., 2023). Additionally, patients treated with Kadcyla may develop resistance because its payload, DM1, is a substrate of multiple drug-resistant transporters (Takegawa et al., 2017; Hunter et al., 2020; Chen et al., 2023).

Enhertu, a highly successful ADC in clinical practice, is currently used to treat various oncological indications, including HER2-positive breast cancer, non-small cell lung cancer, and gastric or gastroesophageal junction adenocarcinoma, all of which have shown promising therapeutic efficacy (Narayan et al., 2021; Balamkundu and Liu, 2023). Interstitial pneumonia-related adverse effects represent a major identifiable risk in its clinical application (Abuhelwa et al., 2022; Nguyen et al., 2023). Prophylactic medications are used to manage drug-induced interstitial pneumonia. Patients with mild disease (up to grade 1), must temporarily undergo administered corticosteroid therapy. Conversely, for those with grade 2 or higher interstitial pneumonia (IDH), drug administration is permanently discontinued (FDA, 2024).

Previous studies have demonstrated a close relationship between the efficacy and toxicity of ADCs and their payloads (Nguyen et al., 2023). The conjugation of small-molecule toxins to antibodies alters their distribution and metabolism, potentially resulting in different efficacy and toxicity profiles compared to the original small molecules. However, the payload remains a key determinant of ADC efficacy and toxicity. With accumulating research, the toxicity of some ADCs is found to be similar to that of the small molecules (Nguyen et al., 2023).

Therefore, exploring new small-molecule toxins and investigating their antitumor activity and toxicity profiles is crucial in ADC development for addressing clinical relapse, resistance, and the varying therapeutic needs of patients.

This study explored a new type of small-molecule toxin, tubulysin, with the aim of achieving different pharmacological and toxicity outcomes. Tubulysin is a subclass of microtubule protein inhibitor molecules that prevent tumor cell proliferation by stopping the polymerization of microtubule proteins (Sasse et al., 2000; Khalil et al., 2006). The Tubulysin class of molecules is a non-multidrug-resistant protein substrate that can be used to combat a wide range of drug resistance (Murray Bc and Fecik, 2015; Stark and Assaraf, 2017; Courter et al., 2020). Several ADCs that utilize tubulysine as the payload have early development data available for review (Murray Bc and Fecik, 2015; Burke et al., 2016). Among these, MEDI4276 has been investigated in patients with HER2-positive advanced breast or gastric cancer, and its primary therapeutic efficacy has been demonstrated. Hepatotoxicity is considered the major dose-limiting toxicity and restricts its therapeutic potential, resulting in the discontinuation of clinical trials (Pegram et al., 2021).

Tub114, a Tubulysin B analog with stable hydrophilic linker, was developed to mitigate hepatotoxicity of this series of small molecules. DX126-262, a novel antibody-drug conjugate, was constructed by conjugating Tub114 with the anti-HER2 monoclonal antibody DX-CHO9. Comprehensive investigations have been conducted to evaluate its pharmacological profile, including its pharmacodynamics, toxicology, and pharmacokinetics.

DX126-262 was also assessed in comparative experiments against the marketed anti-HER2 ADC drugs Kadcyla and Enhertu to evaluate its pharmacological advantages. The results demonstrated that DX126-262 exhibits superior antitumor efficacy compared to Kadcyla and comparable efficacy to Enhertu, along with a favorable toxicity profile.

## Materials and methods

**FIGURE 1:**
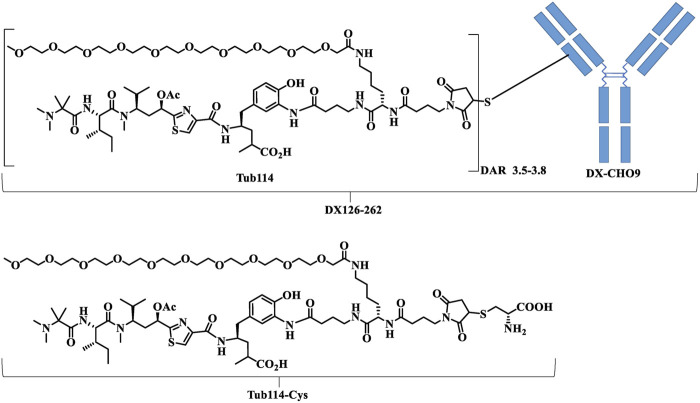
The chemical structures of DX126-262. DX-CHO9 is the monoclonal antibody used in the antibody-drug conjugate (DX126-262). Tub114 is the small molecule linked to the antibody with a DAR of 3.5–3.8. Tub114-cys is the primary metabolite of DX126-262 identified in animal plasma.

Kadcyla^®^ (Roche), Herceptin^®^ (Roche) were purchased from a local pharmacy. DX126-262 is a conjugate of Tub114 and recombinant humanized anti-HER2 monoclonal antibody (DX-CHO9, IgG1) with drug antibody ratio of 3.5–3.8 (Figure 1). Tub114-cys was quantitatively synthesized via the Michael addition of Tub114 with cysteine at pH 6.0–7.5 buffer. Tub114-cys was also the major metabolite of DX126-262 detected in animal plasma. The preparations of DX126-262, DX-CHO9, Tub114 and Tub114-cys, particularly structure–activity relationship (SAR) study of Tub114, will be published in a separate paper.

The BT-474 (RRID: CVCL_0532), SK-BR-3 (RRID: CVCL_0033), NCI-N87 (RRID: CVCL_1603), and MDA-MB-468 (RRID: CVCL_0419) tumor cell lines were obtained from ATCC. BT-474 and SK-BR-3 are human breast cancer cell lines that are HER2-positive. NCI-N87 is an HER2-positive human gastric cancer cell line. MDA-MB-468 is an HER2-negative human breast cancer cell line. These cell lines were cultured in media recommended by ATCC.

BALB/c nude mice (RRID: IMSR_CRL:022, 4–5 weeks) and ICR mice (RRID: IMSR_CRL:194, 7–8 weeks) were purchased from Beijing Vital River Laboratory Animal Technology Co., Ltd. SD rats (RRID: RGD_734476, 6–8 weeks) were purchased from Shanghai Sino-British SIPPR/BK Lab Animal Ltd. Cynomolgus monkeys (approximately 3 years old) were purchased from Guangxi Guidong Primate Development Company, Ltd.

All animal experiments in this study were performed in accordance with the guidelines approved by the Institutional Animal Care and Use Committee (IACUC) of Medicilon (Shanghai, China) or MSLT Biotech (Hangzhou, China), following the guidelines of the Association for Assessment and Accreditation of Laboratory Animal Care.

### *In vitro* target-dependent binding to HER2-positive and HER2-negative cancer cells

BT-474, SK-BR-3, and MDA-MB-468 cells were cultured to the exponential growth phase, detached with 0.25% trypsin, and resuspended in 2% FBS-PBS. A total of 200 μL of the cell suspension (5 × 10^4^ cells per well) was seeded into 96-well plates, centrifuged at 4°C, 2,000 rpm for 1 min, and the supernatant was carefully aspirated. DX-CHO9 and Herceptin were diluted in 2% FBS-PBS to final concentrations ranging from 50 to 0.0005 μg/mL. Two hundred microliters of each dilution was added to the wells, and the plates were incubated at 4°C for 100 min. The plates were then centrifuged at 4°C, 2,000 rpm for 1 min, followed by two washes with 2% FBS-PBS. Subsequently, a PE-labeled secondary anti-human IgG antibody (200 μL per well; Santa Cruz Biotechnology) was added, and the plates were incubated at 4°C for 40 min. After two additional washes with 2% FBS-PBS, fluorescence signals were detected using a flow cytometer (Guava, Merck Millipore). Data analysis was conducted using Guava Soft 3.1.1 software.

### *In vitro* endocytosis into HER2-positive cancer cells

BT-474 and SK-BR-3 cells were cultured to the exponential growth phase, harvested, and washed with pre-cooled PBS. For each sample, 1 × 10^5^ cells were incubated on ice for 1 h with 40 μg/mL of DX126-262, DX-CHO9, Kadcyla, or Herceptin to allow antibody binding, followed by washing to remove unbound antibodies. Alexa Fluor 488-conjugated goat anti-human IgG (H + L) secondary antibody (10 μg/mL; Invitrogen, Shanghai, China) was subsequently added, and the cells were incubated on ice for 30 min. After additional washes, the cells were resuspended in pre-cooled PBS, divided into five aliquots, and incubated at 37°C for 0, 10, 30, 90, or 120 min. At each time point, cells were further split into two sub-aliquots: one treated with Trypan Blue to quench extracellular fluorescence and the other with PBS as an unquenched control. Fluorescence intensity was measured using a flow cytometer (Guava, Merck Millipore). The percentage of endocytosis was calculated as the ratio of quenched to unquenched fluorescence multiplied by 100%.

### *In vitro* inhibition of proliferation of HER2-positive cancer cells

BT-474, NCI-N87, SK-BR-3, and MDA-MB-468 cell lines were cultured for exponential growth, detached, and washed. Cells were adjusted to 3.3 × 10^4^ cells/mL and 180 μL (6 × 10³ cells) were seeded per well in 96-well plates, with a blank control included. The plates were then incubated at 37°C for 24 h. DX126-262, Kadcyla, and Paclitaxel were prepared in complete medium at 100 μg/mL and serially diluted to final concentrations ranging from 100,000–1.28 ng/mL. Two hundred microliters of each dilution were added to the wells in triplicate and incubated at 37°C for 120 h. Following incubation, “5× EZMTT” (JNF Biotech Company) solution was prepared and 20 μL was added to each well. The plates were incubated for 1–3 h at 37°C. Absorbance was measured at 450 nm with a reference wavelength of 620 nm using a microplate reader (Molecular Devices, San Jose, CA, United States). EC_50_ values were calculated using the built-in software.

### *In vivo* efficacy studies

BT-474, NCI-N87, and SK-OV-3 cell lines were established by subcutaneously injecting 6 × 10^6^ tumor cells in a volume of 100 μL with Matrigel/PBS (1:1) into female BALB/c nude mice. In the BT-474 model, animals were supplemented with Estradiol Benzoate (Shusheng, Zhejiang) twice per week via S.C. injection starting 3 days prior to cell inoculation and continuing throughout the experimental period. The dose of Estradiol Benzoate used was 40 μg/20 μL/mouse/injection. The tumors were measured twice a week using a caliper in two dimensions: length (a) and width (b). Tumor volume (TV) was calculated as follows: Tumor Volume (mm^3^) = 0.5 × a (mm) × b (mm)^2^. The relative tumor growth rate (T/C) was calculated as follows: T/C (%) = T_RTV_/C_RTV_ × 100%, where T_RTV_ is the relative mean tumor volume of the treated group (T) and C_RTV_ of the PBS group (C).

High-, medium- and low-dose DX126-262 treated groups were established in all antitumor studies to evaluate dose-dependent relationship. These doses were 10, 5, and 2.5 mg/kg in the BT-474; 12, 8, and 4 mg/kg in NCI-N87; and 16, 8, and 4 mg/kg in SK-OV-3 tumor models, respectively. DX-CHO9 and Tub114-cys of the same molar doses as in the high-dose DX126-262 groups were administered either separately or sequentially to separate groups in each study.

In the BT-474 model, the antitumor effects between DX126-262 and Kadcyla were compared at the same dose levels. DX-CHO9 and Herceptin were evaluated at a dose of 10 mg/kg to differentiate their therapeutic contributions in the respective ADC groups.

The antitumor efficacies of DX126-262 and Enhertu were evaluated in the NCI-N87 model at doses of 6 mg/kg and 1.5 mg/kg. All experimental conditions, tumor measurements, and efficacy evolution were the same as those described above.

### *In vivo* monkey pharmacokinetic studies

Single-dose pharmacokinetic (PK) evaluations were performed at 3, 10, and 30 mg/kg DX126-262 in cynomolgus monkeys. Animals (n = 6 in each group; 3 males and 3 females) were not fasted before blood collection. About 1.2 mL blood sample was collected via the femoral vein at each of the 16 time points from pre-dosing (t = 0) to 41 days post-dosing. Total antibodies, including drug-conjugated and naked antibodies, were analyzed using the HER2 antigen (Shanghai Jinan, CP69) capture ELISA method with a lower limit of quantification (LLOQ) 1.25 ng/mL. Drug-conjugated antibodies (ADC) were analyzed using an anti-Tub114 monoclonal antibody (Hangzhou DAC Biotech. Batch No: 2017082501) ELISA with an LLOQ of 1.25 ng/mL. Plasma-free Tub114-cys was determined by LC-MS/MS with an LLOQ of 2 ng/mL. All three analytes were determined for blood samples collected at the same time points. A separate group received Tub114-cys at a dose of 1 mg/kg and the plasma samples were collected at 13 time points from pre-dosing up to 24 h post-dosing to measure the plasma concentration of Tub114-cys. Concentration-time data was analyzed using a non-compartmental model (WinNonlin, RRID:SCR_024504, Pharsight, United States) to generate PK parameters, such as plasma maximum concentration (C_max_), clearance (CL), area under the concentration-time curve from time 0 to infinity (AUC_inf_), elimination half-life (t_1/2_), and distribution volume at steady-state (V_ss_).

### Acute toxicity studies in ICR mice

Acute toxicities of DX126-262 were compared with those of Kadcyla in female ICR mice following a single intravenous injection at two dose levels. Saline (control), 75 and 150 mg/kg of DX126-262, and Kadcyla were administered to separate groups (n = 10). The study was terminated after 12 days of dosing. Body weights were monitored daily. The serum enzyme activities of AST and ALT were measured on day 5 and 12 after dosing. Half of the mice in each group (n = 5) were euthanized on day 5 and 12. The hearts, livers, spleens, lungs, kidneys and brains were collected and weighed. Pathological examinations using hematoxylin and eosin (HE) staining were conducted on selected organs and tissues, particularly those showing significant weight changes or macroscopic abnormalities, such as the livers.

### Repeated-dose toxicity studies in SD rats

In repeated-dose toxicology studies, SD rats were used to estimate the safety and toxicokinetic profile of DX126-262. Fifty SD rats from six groups (five animals/sex/group) received five doses (i.v., Q3W × 5) of vehicle control (0 mg/kg), DX126-262 (30, 100, or 200 mg/kg), or Tub114-cys (8 mg/kg). Thirty-six animals (three animals/sex/group) were necropsied at the end of the dosing phase (day 89), and the remaining twenty-four animals (two animals/sex/group) were necropsied at the end of the recovery phase (day 141). Clinical observations including body weight, food consumption, and clinical pathology were monitored throughout the study. Macroscopic and microscopic evaluations of the selected tissues were performed.

### Repeated-dose toxicity studies in monkeys

In repeated-dose toxicology studies, cynomolgus monkeys were used to estimate the safety and toxicokinetic profile of DX126-262. Sixty cynomolgus monkeys from six groups (five animals/sex/group) received five doses (i.v., Q3W × 5) of vehicle control (0 mg/kg), DX126-262 (15, 30, or 60 mg/kg), DX-CHO9 (60 mg/kg), or Tub114-cys (3 mg/kg). Thirty-six animals (three animals/sex/group) were necropsied at the end of the dosing phase (day 89), and the remaining twenty-four animals (two animals/sex/group) were necropsied at the end of the recovery phase (day 141). Group 4 (high-dose DX126-262 group) underwent dose adjustment to 45.0 mg/kg at the third dose due to severe toxicity manifestations, and commenced the recovery period on the second day post-dosing (day 44). Throughout the study, clinical observations such as body weight, food consumption, body temperature, and clinical pathology were continuously monitored. Macroscopic and microscopic evaluations of the selected tissues were performed.

## Results

### *In vitro* target-dependent binding and endocytosis to HER2-positive cancer cells

The affinity of DX-CHO9 for HER2-positive cells was comparable to that of Herceptin, with similar K_d_ values observed for DX-CHO9, DX126-262, and Herceptin in both BT-474 and SK-BR-3 cell lines. None of these antibodies bound to HER2-negative MDA-MB-468 cells. Endocytosis studies showed that, DX126-262, Kadcyla, DX-CHO9, and Herceptin had comparable endocytosis efficiencies in HER2-positive BT-474 cells, with maximum rates of approximately 30%–40% within 30–90 min. The endocytosis efficiencies of DX-CHO9 and Herceptin were similar, and conjugation with Tub114 or DM1 did not significantly affect the endocytosis efficiencies of DX126-262 and Kadcyla in either BT-474 or SK-BR-3 cell lines (Supplementary Tables S1, S2; Supplementary Figure S1).

### *In vitro* inhibition of HER2-positive cancer cell proliferation

**FIGURE 2:**
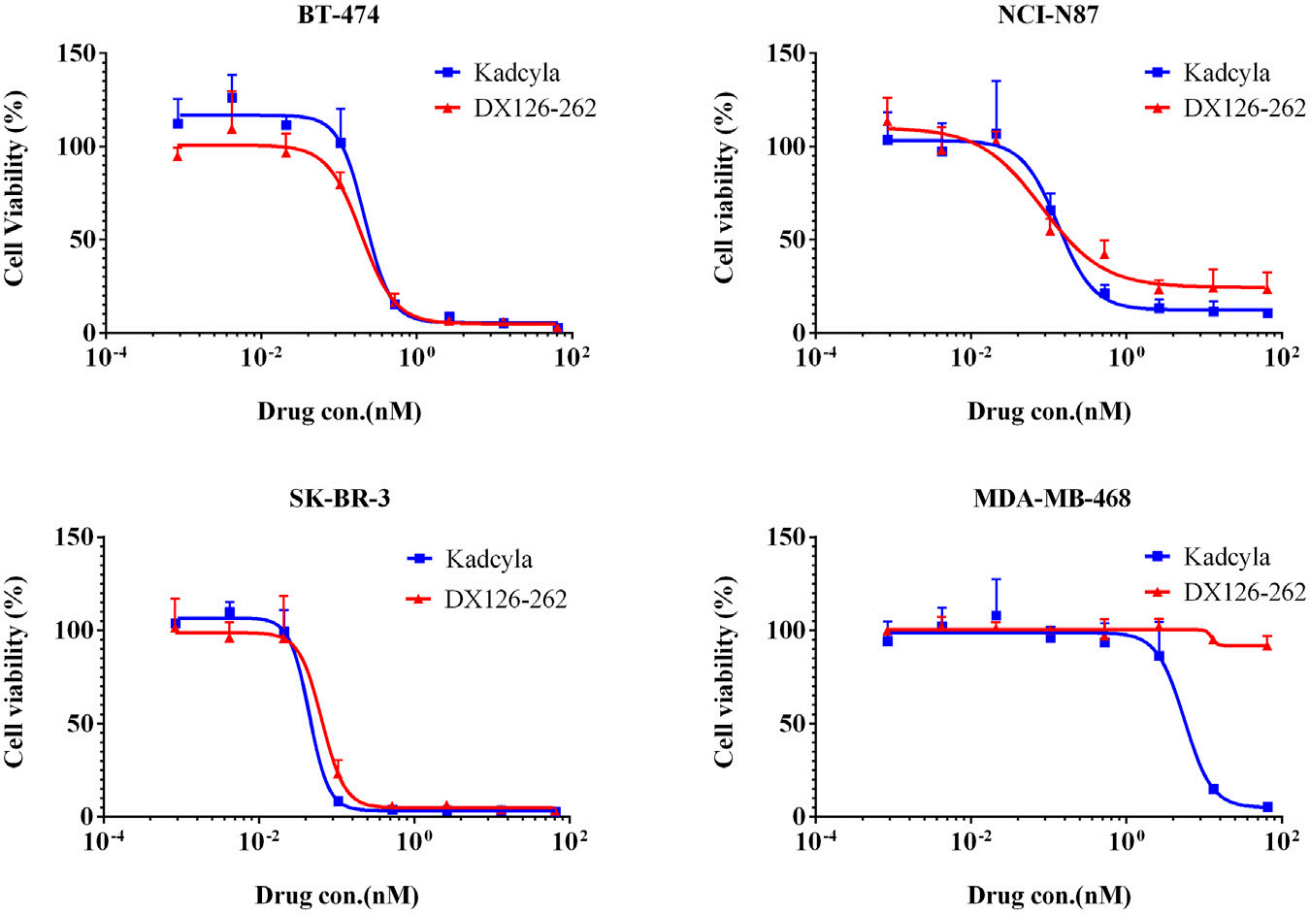
Inhibition of tumor cell proliferation (Cell viability %, mean ± SD of triplicates) plotted as a function of test article concentration (nM). BT-474 (HER2 positive), NCI-N87 (HER2 positive), SK-BR-3 (HER2 positive), MDA-MB-468 (HER2 negtive).

The inhibitory effect on tumor cell proliferation was evaluated by comparing the potency (IC_50_) of DX126-262 and Kadcyla. As shown in Figure 2, DX126-262 exhibited high cytotoxicity against HER2-positive cell lines, with IC_50_ values ranging from 0.06 to 0.19 nM, similar to Kadcyla. Unlike Kadcyla, which showed some inhibitory effects on the HER2-negative cell line MDA-MB-468 at high concentrations, DX126-262 showed no toxicity to this cell line, indicating better target selectivity.

### *In vivo* efficacy studies

**FIGURE 3:**
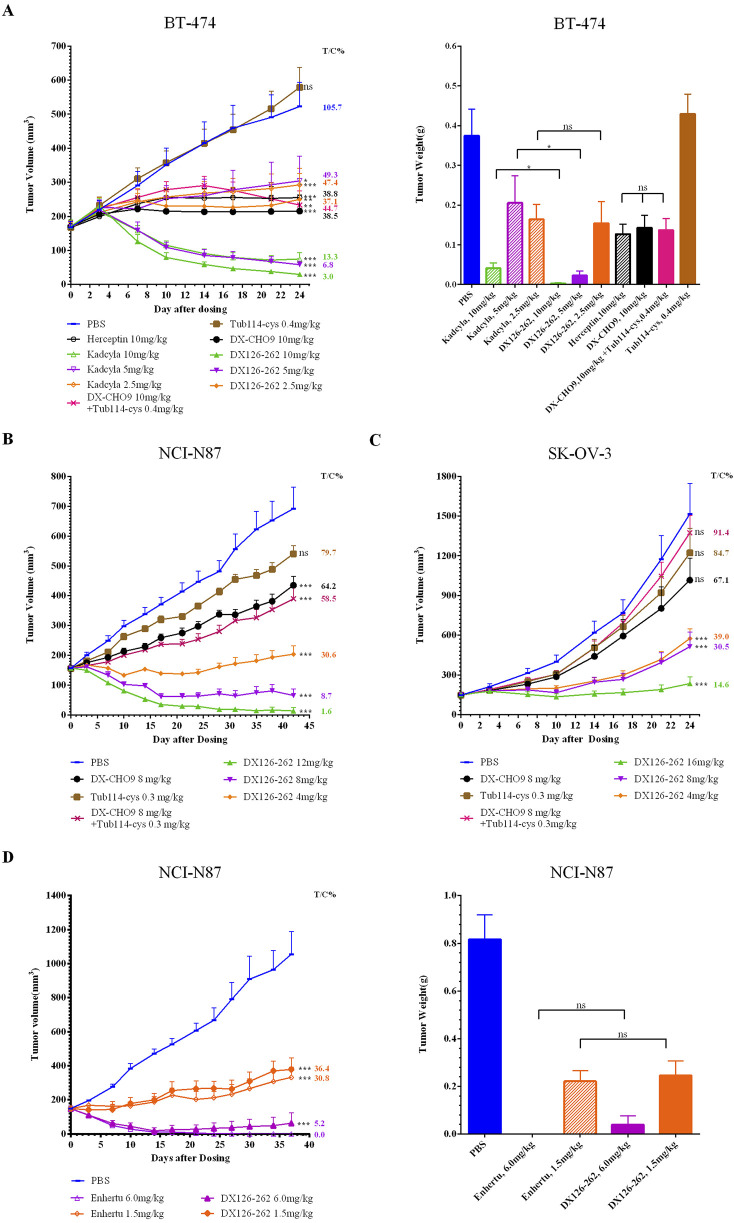
Tumor volumes (mm³) were measured biweekly and plotted as a function of time (days). Tumor weights (g) were recorded post-sacrifice in female BALB/c nude mice bearing subcutaneous xenografts of HER2-positive cancers [n = 10 for **(A–C)**; n = 5 for **(D)**; data presented as mean ± SEM]. **(A)** BT-474 human breast cancer xenografts. Tumor weights were recorded on day 24 post-sacrifice. **(B)** NCI-N87 human gastric cancer xenografts. **(C)** SK-OV-3 drug-resistant human ovarian cancer xenografts. **(D)** NCI-N87 human gastric cancer xenografts. Tumor weights were recorded on day 37 post-sacrifice. One-way analysis of variances (ANOVA) was used. Statistical differences were significant at *p < 0.05 and very significant at **p < 0.01, ***p < 0.001.

In *in vivo* efficacy studies, the antitumor effect of DX126-262 was measurable within 2–4 days, with the maximum activity observed 10–14 days after a single intravenous injection. Significant tumor growth inhibition was observed at the end of the study (approximately 24–42 days post-dosing). DX126-262 demonstrated significant dose-dependent antitumor efficacy across all three tumor models (Figures 3A–C), with T/C% ≤ 40% and *p* ≤ 0.05.

Comparison of DX126-262 with DX-CHO9 and Tub114-cys groups indicated that in the BT-474 model, DX-CHO9 administered alone produced a significant antitumor effect (T/C% was 38.5%, *p* < 0.01), while Tub114-cys had no antitumor effect. Tub114-cys followed by DX-CHO9 administration did not increase the antitumor effect of DX-CHO9. Furthermore, no statistically significant differences were observed in T/C% or tumor weights between the DX-CHO9 and Herceptin groups (38.5% vs. 38.8%, respectively).

In the BT-474 model (Figure 3A), DX126-262 demonstrated superior efficacy compared with Kadcyla at all tested doses. At the high dose, T/C% was 13.3% vs. 3.0%; at the medium dose, T/C% was 49.3% vs. 6.8%; and at the low dose, T/C% was 47.4% vs. 37.1%, respectively. Statistically significant differences (*p* < 0.05) in tumor volumes were observed at the high and medium doses between the DX126-262 and Kadcyla groups. The antitumor effect of 5 mg/kg DX126-262 was better than that of 10 mg/kg Kadcyla, and of 2.5 mg/kg DX126-262 was better than that of 5 mg/kg Kadcyla.

DX126-262 demonstrated comparable efficacy to Enhertu at both high and low doses in the NCI-N87 model (Figure 3D). At a dose of 1.5 mg/kg, DX126-262 exhibited T/C% values of 36.4% compared with 30.8% for Enhertu. At a dose of 6 mg/kg, both DX126-262 and Enhertu induced tumor disappearance. Enhertu achieved complete tumor regression in five out of five animals, while DX126-262 achieved this score in three out of five animals, yielding T/C% values of 0% and 5.2%, respectively.

These observations indicate that DX126-262 exhibited efficacy at least twice of Kadcyla at equivalent dose levels. Moreover, Enhertu demonstrated slightly greater efficacy than DX126-262, although the difference was not statistically significant.

### *In vivo* pharmacokinetic study after single IV injection in cynomolgus monkey

**FIGURE 4:**
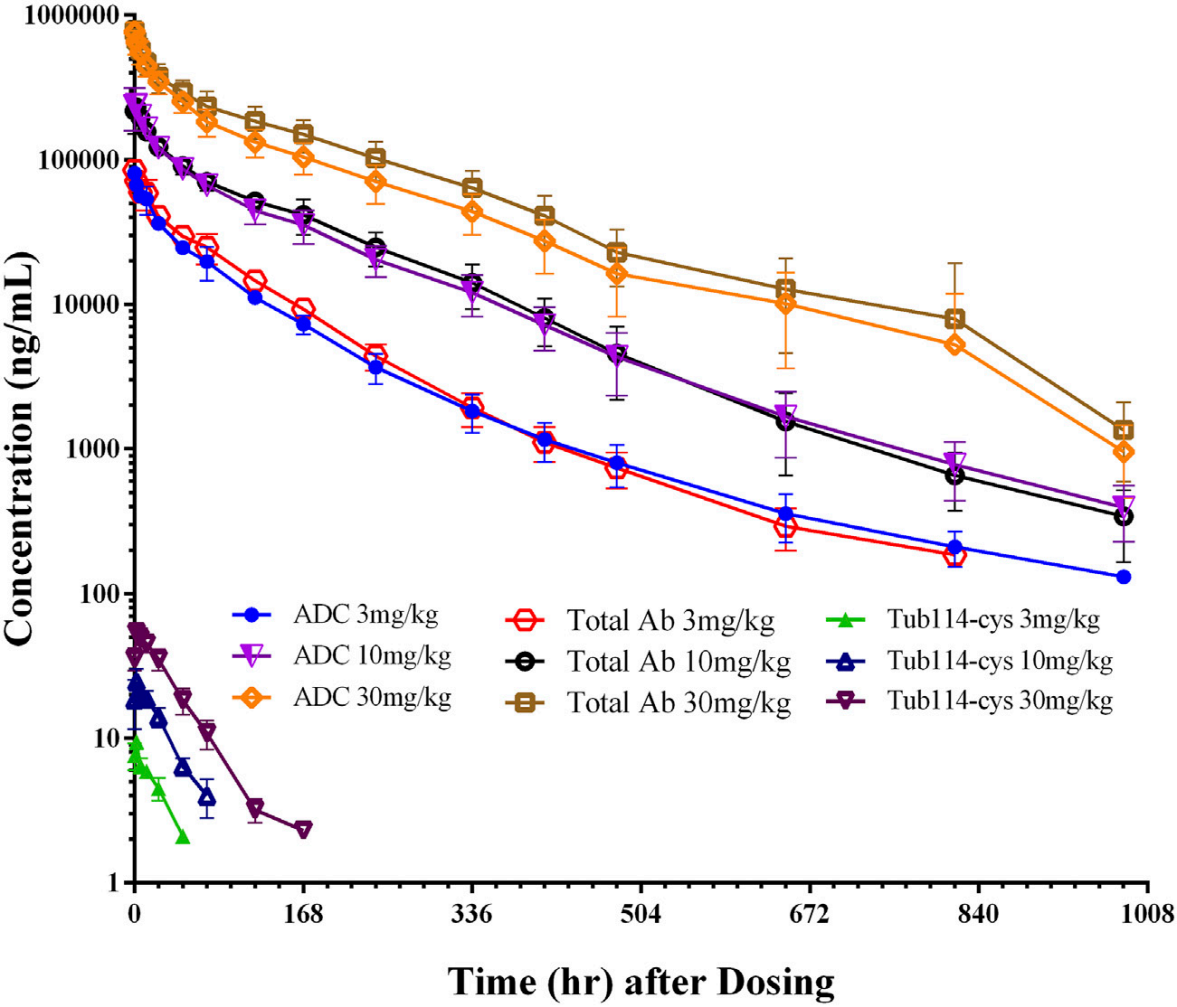
Concentration (μg/mL)-time (hour) curves of DX126-262 (intact ADC), total antibody (total Ab with and without Tub114-cys) and free Tub114-cys in plasma after a single intravenous injection of DX126-262 at 3, 10, and 30 mg/kg.

In the single-dose pharmacokinetic study, DX126-262 was administered to cynomolgus monkeys at three doses (3, 10, and 30 mg/kg). The plasma concentration-time curves are shown in Figure 4. These curves indicate that, following dosing, the levels of both total antibody and ADC increased similarly with escalated doses. The distribution phase lasted for approximately 24 h, and the elimination phase continued until the last time point (41 days post-dosing). Free Tub114-cys was only detectable in the early stage (2–7 days, depending on the dose) after dosing and thereafter below the lower limit of quantitation. As the doses of DX126-262 increased, the Tub114-cys concentration also increased, to be detected with prolonged time period.

Plasma concentration data following the administration of DX126-262 was analyzed using a non-compartmental model, and the primary PK parameters are listed in Table 1. There were no significant differences between male and female (data not shown). The total antibody clearance rate (11.9 mL/day/kg) was slightly lower than that of ADC (13.9 mL/day/kg). The total antibody half-life (approximately 4.9 days) was similar to that of ADC (approximately 5.3 days). At steady state, the distribution volume of the total antibody (67.6 mL/kg) was also close to that of the ADC (77.0 mL/kg), which is equivalent to blood volume per kg of body weight.

**TABLE 1:** Pharmacokinetic parameters after single administration of DX126-262 in cynomolgus monkey. (Mean ± SD).

Analyte	Dose	C_max_	AUC _inf_	T_1/2_	CL	V_ss_
	(mg/kg)	(μg/mL)	(day•μg/mL)	(day)	(mL/day/kg)	(mL/kg)
DX126-262	3	80.6 ± 8.1	196.7 ± 22.9	6.1 ± 1.0	15.4 ± 1.8	71.1 ± 3.4
	10	264.4 ± 30.6	775.1 ± 119.5	5.1 ± 0.5	13.2 ± 2.4	72.8 ± 3.3
	30	766.3 ± 126.4	2,391.7 ± 474.7	4.8 ± 0.4	13.1 ± 3.1	87.2 ± 19.2
Total antibody	3	85.0 ± 8.8	229.1 ± 23.2	5.4 ± 0.6	13.2 ± 1.3	57.9 ± 3.2
	10	244.7 ± 29.9	833.3 ± 136.0	4.6 ± 0.4	12.3 ± 2.2	69.4 ± 4.0
	30	775.4 ± 129	3,056.5 ± 680.9	4.8 ± 0.5	10.3 ± 2.7	75.7 ± 19.1
	(mg/kg)	(ng/mL)	(ng•hr/mL)	(hr)		
Tub114-cys	3	9.5 ± 1.1	519.2 ± 381.9	51.4 ± 47.1		
	10	25.0 ± 5.2	966.4 ± 115.4	26.6 ± 6.6		
	30	54.3 ± 5.7	2,505.7 ± 419.6	28.1 ± 2.7		

Pharmacokinetic parameters for the group administered 1 mg/kg of Tub114-cys alone are listed in Table 2.

**TABLE 2:** Pharmacokinetic parameters after single administration of Tu114-cys in cynomolgus monkey. (Mean ± SD).

Analyte	Dose (mg/kg)	C_max_ (ng/mL)	AUC_inf_ (ng•hr/mL)	T_1/2_ (hr)	CL (mL/hr/kg)	V_ss_ (mL/kg)
Tub114-cys	1	4,155.8 ± 464.2	4,501.5 ± 657.9	1.2 ± 0.2	226.3 ± 34.7	253.8 ± 30.7

### Acute toxicity studies in ICR mice

Acute toxicities of DX126-262 were estimated in female ICR mice after a single intravenous injection of 75 mg/kg or 150 mg/kg, approximately 15–20 times the effective antitumor dose. The toxicity profile of DX126-262 was compared side-by-side with that of Kadcyla (n = 10 in each group). All the test animals survived at the end of the study period.

**FIGURE 5:**
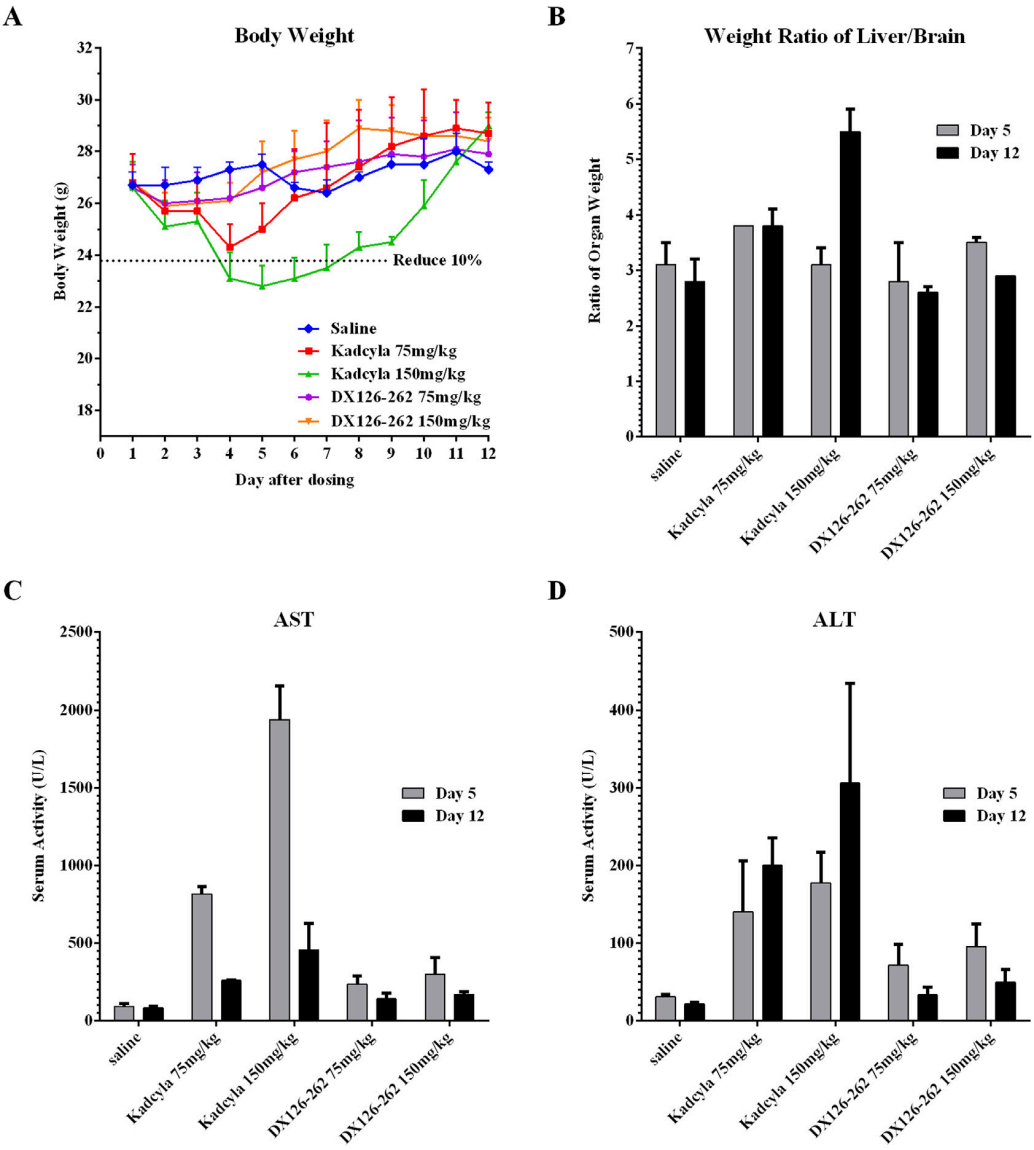
Comparisons of acute toxicities (mean ± SD, n = 10) in ICR mice after a single intravenous injection of DX126-262 or Kadcyla at the same dose levels of 75 and 150 mg/kg. **(A)** Body weights (g) measured daily post-dosing. **(B)** The ratios of liver/brain weights on day 5 and 12 post-dosing. **(C)** Plasma ALT activities (IU/L) on day 5 and 12 post-dosing. **(D)** Plasma AST (IU/L) activities on day 5 and 12 post-dosing.

In the 75 mg/kg Kadcyla-treated group, maximal body weight reduction (−9.3%) occurred on day 4, while the 150 mg/kg group showed a −14.3% reduction on day 5, indicating dose-dependent toxicity. The DX126-262 groups exhibited minimal weight changes (with maximum decreases of −1.5% and −2.6%, respectively) at both doses, suggesting better tolerance (Figure 5A).

At 75 and 150 mg/kg dose levels of Kadcyla, serum AST activity increased by 8.8-fold and 20.8-fold on day 5 and by 3.1-fold and 5.5-fold on day 12, respectively. At 75 and 150 mg/kg dose levels of DX126-262, serum AST activity increased by 2.5-fold and 3.2-fold on day 5, and 1.7-fold and 2.0-fold on day 12 (Figure 5C). At 75 and 150 mg/kg dose levels of Kadcyla, serum ALT activity increased by 4.6-fold and 5.8-fold on day 5 and by 9.4-fold and 14.3-fold on day 12. At dose levels of 75 and 150 mg/kg, DX126-262 serum ALT activity increased by 2.3-fold and 3.1-fold on day 5 and by 1.6-fold and 2.3-fold on day 12 (Figure 5D).

At high doses, both DX126-262 and Kadcyla caused increases in hepatic AST and ALT, with DX126-262 inducing a milder degree of liver damage than Kadcyla on day 5. At the end of the study (day 12), hepatic damage in both DX126-262 groups was recovered substantially, in contrast, hepatic damage was continuous and exaggerated on day 12 in the Kadcyla groups as indicated by the significant increases in hepatic enzyme activities.

At 75 mg/kg and 150 mg/kg dose levels of Kadcyla, the percentages of liver/brain weights increased by 22.6% and 0% on day 5, and by 35.7% and 96.4% on day 12. Significant increases of liver weights in both 75 mg/kg and 150 mg/kg Kadcyla groups were observed, with the exception of 150 mg/kg on day 5, which was probably due to massive and severe damage to the liver, as indicated by pathologic examination (see below). However, at 75 mg/kg and 150 mg/kg dose levels of DX126-262, the percentages of liver/brain weights did not change significantly, with variations of below 15% (Figure 5B).

**FIGURE 6:**
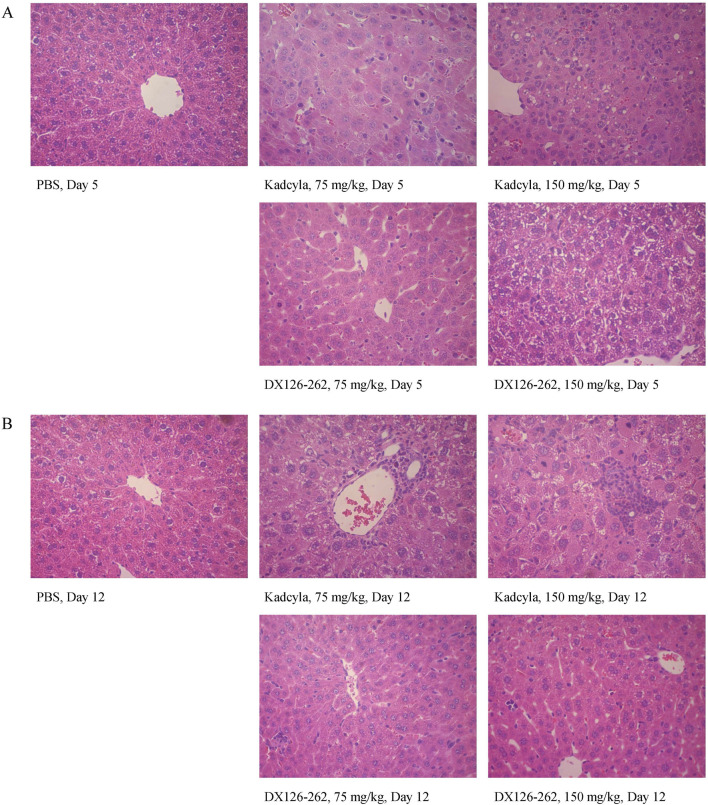
Liver pathologic profiles (H&E staining: ×40) in ICR mice on day 5 **(A)** and day 12 **(B)** after a single intravenous injection of PBS, 75 and 150 mg/kg of DX126-262 or Kadcyla, respectively.

The histopathological examination was consistent with the above observations. After dosing with 75 mg/kg of Kadcyla, on day 5 (Figure 6A), the major hepatic pathological finding was swelling of hepatocytes. After dosing with 150 mg/kg of Kadcyla, on day 5, focal necrosis, vacuolar degeneration of hepatocytes, and infiltration of inflammatory cells were the main pathological observations. After dosing with 75 mg/kg of DX126-262, on day 5, the hepatic pathological findings were mild swelling of hepatocytes and an approximately normal structure of hepatic lobules. After dosing with 150 mg/kg of DX126-262, on day 5, vacuolar degeneration of hepatocytes without focal necrosis and ambiguous hepatic lobules were the main pathologic findings.

On day 12 after dosing with 75 mg/kg of Kadcyla (Figure 6B), mildly swollen hepatocytes, vacuolization around the nucleus, and numerous inflammatory cells surrounding the bile tubules and portal venules were observed. On day 12 after dosing with 150 mg/kg of Kadcyla, the hepatic pathological findings included focal necrosis with infiltration of many inflammatory cells, vacuolar degeneration of hepatocytes, and Kupffer’s cell hypertrophy. On day 12 after dosing with 75 mg/kg of DX126-262, a relatively normal liver structure was observed. On day 12 after dosing with 150 mg/kg of DX126-262, hepatic pathology was relatively normal, with visible spaces in the hepatic sinus and active proliferation of hepatocytes. These pathological findings were consistent with the changes in liver weights and plasma ALT and AST activities, indicating that DX126-262 induced less hepatic toxicity than Kadcyla in ICR mice.

### Repeated-dose toxicity studies in rat

Table 3 summarizes the primary toxicological findings. DX-CHO9 does not bind to HER2 in rats, hence, the toxicities are not target-mediated. Instead, they are off-target and more associated with small molecules. Rats administered with 30 mg/kg of DX126-262 exhibited good tolerance, and all animals survived at the end of the study. The observed toxicities included corneal abnormalities (characterized by mild single-cell necrosis of the epithelium), lung pathology (evidenced by mild to moderate eosinophilic exudate in the alveoli and mild macrophage aggregation), renal complications (indicated by a tubular pattern in the renal tubules), and testicular abnormalities (ranging from mild to severe bilateral varicocele degeneration/atrophy and mild to severe periductal edema around the ductus deferens). In contrast, animals in the 100 and 200 mg/kg groups exhibited more pronounced toxicity, with mild and reversible pathological changes observed in the liver, kidneys, spleen, heart, duodenum, and mammary glands.

**TABLE 3:** Toxicity summary of SD rat repeatedly dosed with DX126-262 and Tub114-cys.

Category	Details
Animal species	SD Rat
Dose	DX126-262: 0,30,100,200 mg/kg; Tub114-cys: 8 mg/kg
Dead/mortality	All animals survived at the end of study
Body weights	Weight loss in the 200 mg/kg DX126-262 group was accompanied by a decrease in food consumption
Hematology	≥30 mg/kg DX126-262: increase in NEUT and MONO
	≥100 mg/kg DX126-262: decrease in RET and increase in WBC
	200 mg/kg DX126-262: decrease in RBC, HGV, PCV and MCHC
	Tub114-cys: decrease in RET
Clinical chemistry	≥100 mg/kg of DX26-262 increase in AST, ALT, GLO and decrease in A/G
	200 mg/kg DX126-262: increases in ALP, TCHO and TG
	Tub114-cys: increases in CREA and TG
Target organs/tissues	Kidneys, liver, lungs, heart, spleen, testicles, breast, eyeball and the duodenum
HNSTD	100 mg/kg

### Repeated-dose toxicity studies in monkey

Cynomolgus monkeys exhibit tissue-specific HER2 expression patterns that are highly similar to those found in humans, and DX-CHO9 binds to cynomolgus and human HER2 with comparable affinities. Therefore, cynomolgus monkeys are regarded as the most appropriate species for assessing the on-target toxicity. Table 4 summarizes the significant toxicity findings.

**TABLE 4:** Toxicity summary of cynomolgus monkeys repeatedly dosed with DX126-262 and Tub114-cys.

Category	Details
Animal species	Cynomolgus monkeys
Dose	DX126-262: 0,10, 30, 60/45 mg/kg; Tub114-cys: 3 mg/kg
Dead/mortality	Weight loss in the 60/45 mg/kg DX126-262 group was accompanied by a decrease in food consumption
Body weights	60/45 mg/kg DX126-262 group showed decline in body weights, accompanied by a decrease in food consumptions
Hematology	≥10 mg/kg DX126-262: increases in NEUT and MONO
	≥30 mg/kg DX126-262: decreases in HGB, HCT and increase in RET
	60 mg/kg DX126-262: increases in WBC
	3 mg/kg Tub114-cys: increases in NEUT and MONO
Clinical chemistry	≥30 mg/kg DX126-262 increase ALP, AST, TG, GLO, TP, GGT, and inversion of A/G
	60 mg/kg DX126-262 increases in TBIL, DBIL, ALT, CREA, UREA, ALB, blood calcium, phosphorus
	Tub114-cys: increases in CREA and TG
Toxic target organs/tissues	≥50 mg/kg: liver, kidneys, lungs, thymus, spleen, lymph nodes
	≥30 mg/kg: liver, kidneys, lungs
HNSTD	30 mg/kg

DX126-262 is well tolerated in cynomolgus monkeys at doses up to 30 mg/kg. The dose of 60 mg/kg resulted in mortality in two animals on day 26 and 28. Therefore, the dose was reduced to 45 mg/kg for the third administration, and animals began the recovery phase on the second day. However, at the end of the study, six of the ten animals in the high-dose group died. Severe clinical signs emerged shortly before death or impending death. Histopathological analysis revealed immune system toxicity, characterized by lymphopenia in the thymus, spleen, and lymph nodes, alongwith necrosis of splenic macrophages, leading to mortality.

At doses of ≥30 mg/kg, DX126-262 predominantly manifested toxicity in high-perfusion organs, specifically affecting the liver (mild to moderate hepatocyte/blastocyte degeneration/necrosis), lungs (mild to moderate increase in alveolar macrophages), and kidneys (mild renal tubular degeneration/necrosis). Additionally, it induced anemia, characterized by reduced red blood cell counts, hemoglobin levels, and erythrocyte volume, but did not impair hematopoiesis in the bone marrow erythrocyte lineage.

At the dose of 10 mg/kg, DX126-262 exhibited relatively mild toxicity, with no significant deviations from the control group and no pathological abnormalities detected.

## Discussion

Tubulysin are potent tubulin-binding antitumor agents. Due to the excellent antitumoral, antimetastatic, antiangiogenic, and anti-multidrug resistance properties, tubulysins are believed to be a promising payload for ADCs.

DX126-262 was generated by conjugating an anti-HER2 monoclonal antibody with a novel tubulysin B analog via a stable thiol-maleimido ether linker containing a hydrophilic branched polyethylene glycol unit. The main structural difference between natural Tubulysin B and the Tubulysin B analog here is that the original 1-methylpiperidine-2- carboxylic group is replaced with a 2-(dimethylamino)-2-methylpropanoic group, in order to reduce the liver toxicity. The branched ethylene glycol unit in the linker is used to maintain the stability of the conjugate in blood circulation and increase hydrophilicity.

*In vitro* evaluations demonstrated that the cell binding and endocytosis rates of DX126-262 and Kadcyla in target-positive tumor cells were comparable. Additionally, DX126-262 showed similar inhibition of HER2-positive cell proliferation as Kadcyla.

*In vivo* efficacy studies performed using three HER2-positive xenograft tumor models indicated that DX126-262 had significant antitumor efficacy in the given dose range in a dose-dependent and target-selective manner in all three tumor models. The maximum antitumor effect was achieved with 5 mg/kg DX126-262 in the BT-474 model. Increasing the dose to 10 mg/kg did not further increase antitumor activity, suggesting a mechanism by which saturation of targets had been reached at the 5 mg/kg level. A similar antitumor activity was observed in the NCI-N87 tumor model at a dose of 8 mg/kg. Notably, in the SK-OV-3 model, a commonly used ovarian cancer model resistant to platinum complexes (Hills et al., 1989), 16 mg/kg of DX126-262 resulted in 14.6% T/C% tumor inhibition lasting for at least 24 days.

DX126-262 was compared with Kadcyla or Enhertu at multiple doses in the BT-474 and NCI-N87 models, respectively. In the BT-474 model, DX126-262 demonstrated greater antitumor effectiveness at half the dose of Kadcyla. In the NCI-N87 model, DX126-262 exhibited antitumor activity comparable to Enhertu at both high and low dose levels. At the high dose of 6 mg/kg, the average tumor volumes showed no significant difference between the two treatments. All tumors in the Enhertu group regressed completely and showed no recurrence until the end of the study. In the DX126-262 group, one animal’s tumor did not completely regress and lost its inhibitory effect after day 15, resulting in a slightly larger average tumor volume than that of in the Enhertu group.

The pharmacokinetic profile of DX126-262 was compared with published data from a single intravenous injection of Kadcyla and Enhertu in cynomolgus monkeys (FDA, 2013; Nagai et al., 2019). Although these three HER2 ADCs exhibited similar pharmacokinetic profiles, each maintained distinct characteristics. Similar to Enhertu, after the administration of DX126-262, the plasma concentrations of ADC and total antibodies were nearly identical at each time point, with no significant differences in distribution volume, half-life (T_1/2_), or exposure levels between the ADC and total antibodies. In contrast, Kadcyla demonstrated notable differences between conjugated and total antibodies, particularly at low doses (3 mg/kg), where the clearance of the conjugated antibody was markedly faster than that of the total antibody (Nagai et al., 2019). Furthermore, following doses of 3, 10, and 30 mg/kg of DX126-262, the C_max_ of Tub114-cys were 5.5, 14.6, and 31.7 nM, while that of DM1 in Kadcyla were 8.0, 28.2, and 90.2 nM (Poon et al., 2013). The concentration of the free payload generated by Kadcyla was 2–3 times higher than that produced by DX126-262. Collectively, these results confirmed that the linker in DX126-262 exhibited greater stability than that in Kadcyla.

A comparative analysis of the small molecules Tub114-cys and Dxd, each administered at a dose of 1 mg/kg, revealed that Tub114-cys has a significantly lower clearance (CL) than Dxd. The clearance of Dxd was comparable to hepatic blood flow in cynomolgus monkeys (Hall et al., 2012), suggesting that Dxd is primarily cleared through hepatic pathways. In contrast, this finding indicates that the liver may not be a major route of clearance for free payload Tub114-cys. Additionally, a comparative evaluation of V_ss_ values revealed that Tub114-cys (253.8 mL/kg) has lower tissue affinity compared to Dxd (832 mL/kg). Although there are no published data on DM1 alone in cynomolgus monkeys, experimental data from rat studies indicate that the V_ss_ of DM1 exceeds the total body water content in rats (668 mL/kg), further suggesting a higher tissue affinity (Pharmaceuticals and Medical Devices Agency, 2013).

Hepatotoxicity has previously been reported as the dose-limiting toxicity of tubulysin (Pegram et al., 2021). In a direct comparison, mice were given high doses of DX126-262, mainly compared with the hepatotoxic drug Kadcyla, to assess the liver toxicity of DX126-262. *In vivo* comparisons of acute toxicities after single intravenous injections of 75 and 150 mg/kg of DX126-262 or Kadcyla were conducted in mice. At equivalent doses, the body weight reduction induced by DX126-262 was less pronounced, and recovery was faster than that induced by Kadcyla. The results of the hepatic aminotransferase assay and histopathological analysis showed that DX126-262 caused less hepatotoxicity than Kadcyla, with better tolerance in animals.

Subsequently, repeated-dose studies in rats and monkeys demonstrated that DX126-262 was well tolerated. In a long-term toxicity study in rats, 200 mg/kg DX126-262 was tolerated, most of the observed toxicities were reversible or animals showed signs of recovery by the end of the recovery period. In contrast, Kadcyla at a dose of 60 mg/kg under a single-dose regimen resulted in animal death (Poon et al., 2013). Since neither DX126-262 nor Kadcyla binds to rat HER2, the observed toxicity was off-target toxicity, suggesting that Tub114-cys is better tolerated than DM1. Hepatic pathological microscopy revealed only slight single-cell necrosis in the medium- and high-dose groups of DX126-262, which had recovered at the end of the recovery period.

In repeated-dose studies conducted in cynomolgus monkeys, all animals tolerated a dose of 30 mg/kg, administered five times. The primary toxicities of DX126-262 were observed in hyperperfused organs such as the livers, lungs, and kidneys, as well as in lymphoid organs, including the thymus, spleens, and lymph nodes, manifesting as marked lymphopenia and macrophage increase or necrosis. These toxicity profiles closely resemble those of Kadcyla. This toxicity distribution pattern was not associated with HER2 expression, possibly because the low HER2 expression in normal tissues, combined with the large size of the antibody, limits tissue penetration, resulting in its predominant presence in the peripheral blood. Additionally, DX126-262 uses a non-cleavable linker, leading to nonspecific uptake by highly perfused organs along with the antibody. The antibody also can bind to immune cells, such as monocyte macrophages, via FcγR, triggering the release of small molecules that induce toxicity in these tissues.

Regarding lung toxicity, DX126-262 caused slight to moderate increase in lung macrophages in several animals from the medium- and high-dose groups. It also induced slight to moderate alveolar edema, with or without slight to moderate increase in alveolar macrophages, during the recovery phase. The analysis of the cause of animal death revealed a mild increase in alveolar macrophages in one case. These results align with observations from Kadcyla and Enhertu, suggesting a potential target-independent uptake of anti-HER2 ADCs by alveolar macrophages (FDA, 2013; Kumagai et al., 2020).

It is widely believed that HER2 ADC-induced interstitial pneumonitis is closely related to small molecules themselves (Skeoch et al., 2018). Possible causes include uptake and degradation of ADCs by target or other tissues, and “killing” or “bystander killing” by the free payload released through degradation or reverse conjugation of ADCs (Kumagai et al., 2020). Regardless of the specific mechanism, the root cause of interstitial pneumonitis is the release of small molecules. Retrospective data suggest that drug-induced interstitial pneumonitis is related to the intrinsic properties of the drug itself. For instance, in the case of HER2 ADC, Enhertu, which utilizes the Dxd molecule, interstitial lung disease (ILD)/pneumonitis was reported in 25 out of 184 patients (13.6%) in a global Phase II study involving HER2-positive metastatic breast cancer patients previously treated with trastuzumab emtansine (Modi et al., 2020). T-DM1, which utilizes the DM1 molecule, has an ILD/pneumonitis incidence of approximately 1% (Montemurro et al., 2019). For ARX788, which uses the MMAF molecule, ILD/pneumonitis was reported in 6 out of 30 (20%) patients in a Phase I trial for advanced gastric and gastroesophageal junction adenocarcinoma (Zhang et al., 2022). Since these ADCs utilize similar antibodies, the significant differences in the incidence of ILD may be attributed to variations in the conjugated small molecules.

Tublysin B analogue used in DX126-262 was modified to increase its hydrophilicity. As a result, the hydrophilic payload molecules Tub114 and Tub114-cys are believed to be unable to cross the cell hydrophobic membranes once being released. In *in vitro* cytotoxicity studies, neither Tub114 nor Tub114-cys could kill tumor cells at the same concentration as the corresponding ADC (Supplementary Figure S2). Furthermore, in the monkey pharmacokinetic study, Tub114-cys exhibited lower tissue affinity than other molecules, leading to reduced tissue diffusion and a lower toxicity impact on surrounding tissues. All studies suggest a potential reduction in the risk of interstitial pneumonitis. Similarly, MEDI4276, which also employs tubulysin as a small molecular payload, did not report any cases of interstitial pneumonitis in a Phase I study (Pegram et al., 2021).

In conclusion, DX126-262 demonstrated superior antitumor efficacy compared to Kadcyla and showed effectiveness comparable to Enhertu. Additionally, it was well-tolerated in animal toxicity studies. The incorporation of a novel hydrophilic Tubulysin B molecule, Tub114, helps to reduce toxicity risks, particularly hepatotoxicity. Preliminary data indicated that tubulysin compounds do not cause ILD/pneumonitis. Given its favorable safety profile and therapeutic efficacy, DX126-262 represents a promising therapeutic option for patients resistant to existing treatments or those seeking safer alternatives.

DX126-262 received approval from the Chinese NMPA and US FDA for Phase I-II clinical trials. In the dose escalation study, ranging from 0.6 to 8.0 mg/kg, no adverse events (AEs) above grade two were observed, and partial responses (PRs) were achieved starting at 3.6 mg/kg. This drug is currently undergoing Phase II clinical evaluation and the clinical data will be published in due course.

## Data Availability

The datasets presented in this study can be found in online repositories. The names of the repository/repositories and accession number(s) can be found in the article/Supplementary Material.

## Supplementary material

SUPPLEMENTARY FIGURE S1 Binding affinity (MFI: Mean Fluorescence Intensity, mean of the triplicates) plotted as a function of test article concentration (μg/mL).

SUPPLEMENTARY FIGURE S2 Inhibition of tumor cell proliferation (Cell viability %, mean ± SD of triplicates) plotted as a function of Tub114 and Tub114-Cys concentrations (nM).
