# Supplementary material for: The development and evaluation of a tublysine-based antibody-drug conjugate with enhanced tumor therapeutic efficacy
Source: Front Pharmacol. 2025 Feb 10;16:1532104. doi: 10.3389/fphar.2025.1532104 (PMC11847841; doi:10.3389/fphar.2025.1532104)
Supplement: Supplementary file 1 [file Table1.docx]

**Table S1.** Binding Affinity data of DX-CHO9 (HER2 antibody), DX126-262 (HER2-ADC) and Herceptin against BT-474 (HER2 positive cell), SK-BR-3 (HER2 positive cell) and MDA-MB-468 (HER2 negative cell).

|  | | | |
| --- | --- | --- | --- |
| Cell line | BT-474 | | |
|  | DX-CHO9 | DX126-262 | Herceptin |
| K_d_(μg/mL) | 0.1399 | 0.2117 | 0.1365 |
| Cell line | SK-BR-3 | | |
|  | DX-CHO9 | DX126-262 | Herceptin |
| K_d_(μg/mL) | 0.0989 | 0.1225 | 0.07867 |
| Cell line | MDA-MB-468 | | |
|  | DX-CHO 9 | DX126-262 | Herceptin |
| K_d_(μg/mL) | 3.65E-05 | 5.74E-05 | 3.53E-06 |
