# Supplementary figures and images for: The development and evaluation of a tublysine-based antibody-drug conjugate with enhanced tumor therapeutic efficacy
Source: Front Pharmacol. 2025 Feb 10;16:1532104. doi: 10.3389/fphar.2025.1532104 (PMC11847841; doi:10.3389/fphar.2025.1532104)

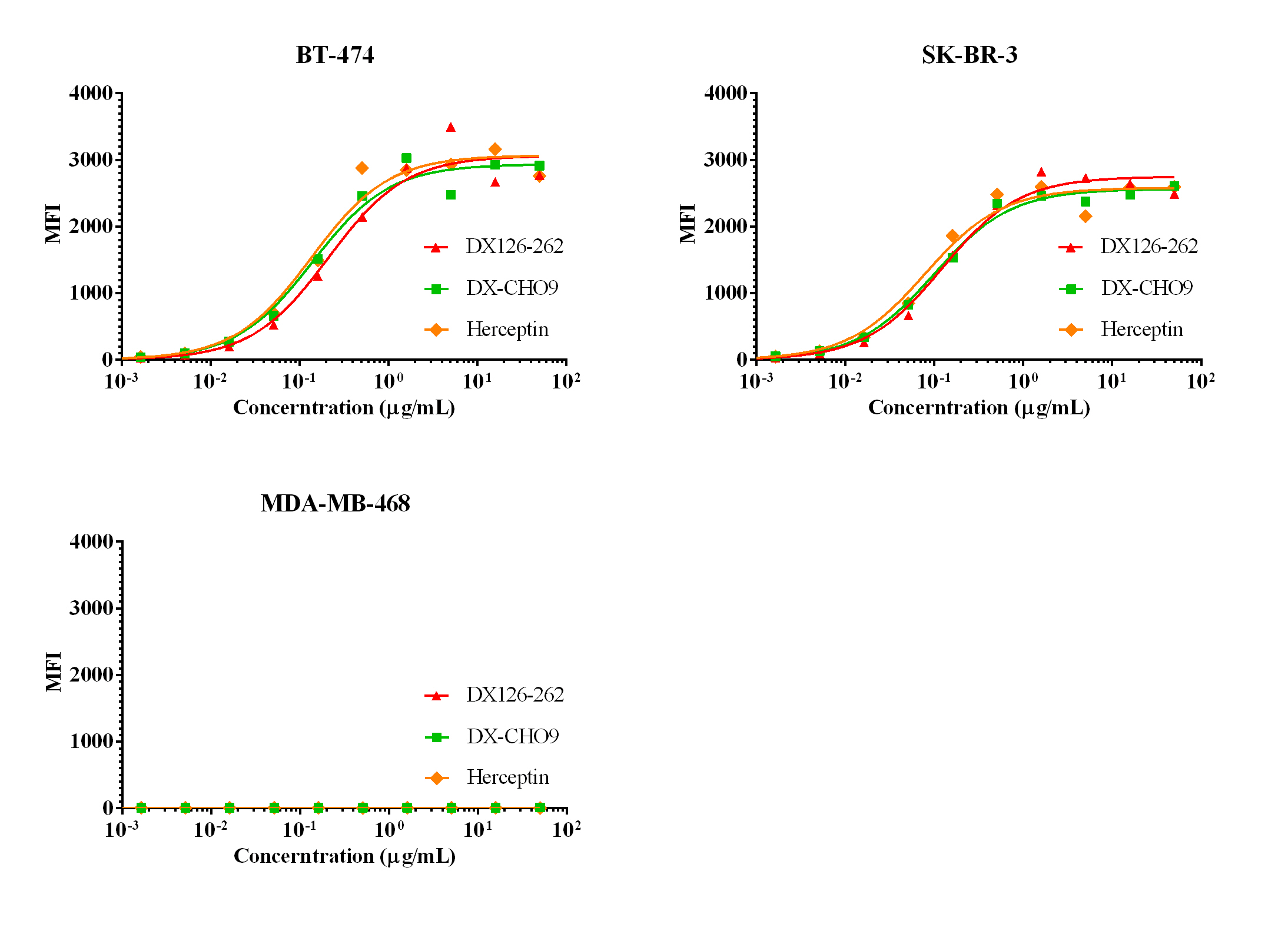

Supplement: Supplementary file 2 [file Image1.jpeg]

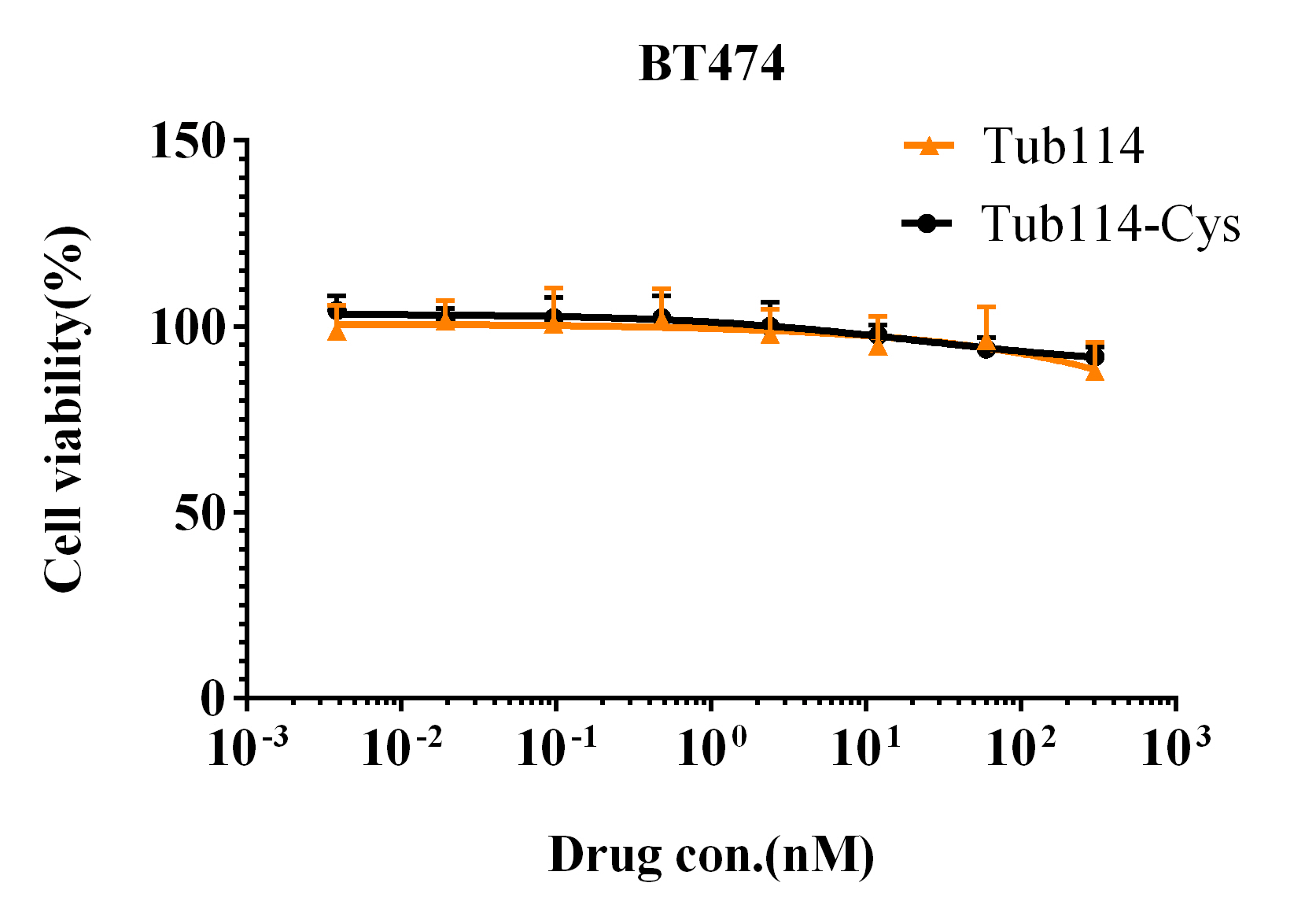

Supplement: Supplementary file 3 [file Image2.jpeg]
