# Supplementary material for: The development and evaluation of a tublysine-based antibody-drug conjugate with enhanced tumor therapeutic efficacy
Source: Front Pharmacol. 2025 Feb 10;16:1532104. doi: 10.3389/fphar.2025.1532104 (PMC11847841; doi:10.3389/fphar.2025.1532104)
Supplement: Supplementary file 4 [file Table2.docx]

**Table S2.** Comparisons of Endocytosis Efficiency (%, Mean of Triplicates) with Incubation Time of DX-CHO9, DX126-262, Herceptin, and Kadcyla in HER2-Positive BT-474 and SK-BR-3 Tumor Cell Lines.

| Cell line | Test article | Incubation time (min) | | | | |
| --- | --- | --- | --- | --- | --- | --- |
|  |  | 0 | 10 | 30 | 90 | 120 |
|  |  | Endocytosis Efficiency (%) | | | | |
| BT-474 | DX-CHO9 | 9.8 | 32.9 | 40 | 44.4 | 42 |
|  | DX126-262 | 11.2 | 31.3 | 37.1 | 43.3 | 40.5 |
|  | Herceptin | 11.3 | 30 | 36 | 41.8 | 39.9 |
|  | Kadcyla | 10.8 | 27.3 | 34.2 | 38.6 | 43.8 |
| SK-BR-3 | DX-CHO9 | 10.4 | 13.5 | 19.9 | 37.1 | 38.4 |
|  | DX126-262 | 9.9 | 12.7 | 18.6 | 33.6 | 35.2 |
|  | Herceptin | 10.1 | 13.6 | 18.8 | 35 | 37.4 |
|  | Kadcyla | 9.3 | 11.8 | 17.4 | 31.6 | 33.4 |
